# Fish oil alleviated high-fat diet–induced non-alcoholic fatty liver disease via regulating hepatic lipids metabolism and metaflammation: a transcriptomic study

**DOI:** 10.1186/s12944-016-0190-y

**Published:** 2016-02-01

**Authors:** Fahu Yuan, Hualin Wang, Yu Tian, Qi Li, Lei He, Na Li, Zhiguo Liu

**Affiliations:** Wuhan Polytechnic University, School of Biology and Pharmaceutical Engineering, Wuhan, Hubei 430023 China; Jianghan University, School of Medicine, Wuhan, China; Department of Blood Transfusion, Tongji Hospital, Tongji Medical College, Huazhong University of Science and Technology, Wuhan, China

**Keywords:** hepatic, high-fat diet, inflammation, lipid metabolism, n-3 PUFA, NAFLD, RNA-Seq

## Abstract

**Background:**

Intake of fish oil rich in n-3 polyunsaturated fatty acids (PUFAs) is believed to be beneficial against development of non-alcoholic fatty liver disease (NAFLD). However, the underlying mechanisms remain unclear. This study was to gain further understanding of the potential mechanisms of the protective effects of fish oil against NAFLD.

**Methods:**

Ten male Sprague–Dawley rats were fed a control diet (CON), a Western style high-fat and high-cholesterol diet (WD), or a WD diet containing fish oil (FOH) for 16 weeks respectively. The development of liver steatosis and fibrosis were verified by histological and biochemical examination. Hepatic transcriptome were extracted for RNA-seq analysis, and particular results were confirmed by real-time polymerase chain reaction (PCR).

**Results:**

The consumption of fish oil significantly ameliorated WD-induced dyslipidemia, transaminase elevation, hepatic steatosis, inflammatory infiltration, and fibrosis. Hepatic RNA-Seq analysis showed that long-term intake of fish oil restored the expression of circadian clock-related genes *per2* and *per3*, which were reduced in WD fed animals. Fish oil consumption also corrected the expression levels of genes involved in fatty acid and cholesterol metabolism, such as *Srebf1*, *Fasn*, *Scd1*, *Insig2*, *Cd36*, *Cyp7a1*, *Abcg5*, *Abcg8* and *Pcsk9*. Moreover, the expression levels of pro-inflammation genes *Mcp1*, *Socs2*, *Sema4a*, and *Cd44* in the FOH group were lower than that of WD group, implying that fish oil protects the liver against WD-induced hepatic inflammation.

**Conclusion:**

The present study demonstrates fish oil protects against WD-induced NALFD via improving lipid metabolism and ameliorating hepatic inflammation. Our findings add to the current understanding on the benefits of n-3 PUFAs against NAFLD.

**Electronic supplementary material:**

The online version of this article (doi:10.1186/s12944-016-0190-y) contains supplementary material, which is available to authorized users.

## Background

Non-alcoholic fatty liver disease (NAFLD) is the most common chronic liver disease in developed countries, and is present in 20-30 % of Western population. Its incidence in the urban population of developing countries such as China has also increased rapidly in the recent decades[1–3]. The typical pathological features of NAFLD include excessive triacylglycerol (TG) accumulation and micro/macrolipid vesiculae formation in the liver. The initial stage of the disease is simple steatosis; approximately 30-40 % of patients progress to non-alcoholic steatohepatitis (NASH) with or without cirrhosis [4–7].

The excessive intake of typical Western-style high-fat, high-cholesterol, and high-sugar diets and lack of appropriate physical exercise are believed to lead to hepatic steatosis; however, the reason for progression of simple steatosis to non-alcoholic steatohepatitis (NASH) is still unclear. Classical “two-hits” hypothesis and modified “multiple-hits” hypothesis suggest that TG accumulation in liver combined with insulin resistance, endoplasmic reticulum stress (ERS), oxidative stress, and hepatic inflammation lead to NASH, hepatocyte death, and liver fibrosis [8, 9]. The saturated free fatty acids (FFAs) and free-cholesterol accumulation in circulation and liver contribute to the progression from simple steatosis to NASH, owing to FFA-induced toll-like receptor (TLR) 4 activation, pro-inflammatory cytokines release, and lipid overload-induced hepatocyte apoptosis, which is also known as lipoapoptosis[10–13]. Diets with high fat content, especially saturated fatty acids, may promote the development of NASH [14, 15].

Conversely, n-3 polyunsaturated fatty acids (PUFAs), such as docosahexaenoic acid (DHA), have been found to decrease liver fat content in children with NAFLD and possess anti-inflammatory effects. Moreover, recent studies indicated the benefits of n-3 PUFAs in lipid metabolism modification, dyslipidemia improvement, and hepatic inflammation mitigation, which may contribute to the amelioration of NAFLD. A latest meta-analysis review presented by Parker et al. [16] showed that dietary n-3 PUFA supplements ameliorated the hepatic steatosis and liver injury in adult NAFLD patients. In the present study, we set up a Western style high-fat and high-cholesterol diet (WD)-induced NAFLD rat model, aim to study the protective effects of fish oil-enriched n-3 PUFAs against NAFLD. In order to get a prospective view of fish oil induced hepatic gene expression change in NAFLD rats, we constructed an RNA-sequencing–based transcriptomic approach to investigate the potential molecular mechanisms.

## Results

### FOH had no effects on bodyweight change compared with WD-fed rats

Male 8–9 weeks SD rats were placed on a normal chow diet (CON), a high-saturated fat and high-cholesterol Western style diet (WD) or a WD diet with 10 % fish oil (w/w) (FOH) for 16 weeks. The rats fed high-calories diets were significantly heavier than those fed CON (*P* < 0.01). On the other hand, fish oil had no significant effects on the bodyweight compared with WD-fed rats (Table 1, Additional file 1: Figure S3).

**Table 1:** Phenotypic comparison of rats fed the CON, WD, or FOH diets for 16 weeks

	CON	WD	FOH
Body weight, g	381 ± 15	478 ± 14^a^	461 ± 15 ^a^
Plasma variables			
Glucose, mmol/L	7.42 ± 0.25	7.77 ± 0.22	7.75 ± 0.26
TG, mmol/L	0.61 ± 0.06	1.16 ± 0.16 ^a^	0.56 ± 0.04^b^
TCh, mmol/L	1.51 ± 0.13	2.87 ± 0.21 ^a^	1.25 ± 0.05 ^b^
HDL-C, mol/L	0.74 ± 0.04	0.58 ± 0.03	0.52 ± 0.02
LDL-C, mmol/L	0.57 ± 0.02	1.8 ± 0.33 ^a^	0.29 ± 0.05 ^b^
NEFA, μmol/L	964.16 ± 65.73	1551.12 ± 88.06 ^a^	1284.85 ± 25.59 ^a b^
ALT, U/L	47.32 ± 1.31	68.4 ± 1.95 ^a^	56.82 ± 1.63 ^a b^
AST, U/L	94.74 ± 2.2	166.98 ± 9.37 ^a^	120.12 ± 4.67 ^ab^
GST, U/L	80 ± 1.78	104.25 ± 4.52 ^a^	81.67 ± 1.21 ^b^
SOD, U/L	175.95 ± 5.23	144.36 ± 5.75 ^a^	161.82 ± 3.13^b^
Liver variables			
Weight, g	13.08 ± 1.38	22.80 ± 4.22 ^a^	21.69 ± 1.89 ^a^
Liver to body weight ratio, %	3.09 ± 0.22	4.79 ± 0.62 ^a^	4.43 ± 0.31 ^a^
TG, μmol/mg protein	0.20 ± 0.05	0.5 ± 0.07 ^a^	0.34 ± 0.10 ^b^
TCh, μmol/mg protein	0.4 ± 0.01	1.19 ± 0.14 ^a^	0.43 ± 0.03 ^b^

TG: triacylglycerol, TCh: total cholesterol, HDL-C: high-density lipoprotein cholesterol, LDL-C: low-density lipoprotein cholesterol, NEFA: non-esterified fatty acid, ALT: alanine aminotransferase, AST: aspartate aminotransferase, GST: glutathione S- transferase, SOD: superoxide dismutase.

Values are expressed as mean ± SD; n = 10 in each treatment group. ^a^ *P* < 0.05 compared with CON, ^b^ *P* < 0.05 compared with WD.

### FOH ameliorated WD-induced hyperlipidemia

The routine blood biochemical tests showed that neither WD nor FOH had affected the fasting blood glucose level compared with low-fat CON. However, compared with the CON group, the plasma TG, total cholesterol (Tch), low-density lipoprotein cholesterol (LDL-C), and non-esterified fatty acid (NEFA) concentration in rats fed WD increased by 90 %, 90 %, 216 %, and 61 %, respectively (*P* < 0.05). The effects of WD on plasma lipid levels were attenuated by fish oil feeding. Compared with the WD group, the plasma TG, TCh, LDL-C, and NEFA concentrations in the FOH group decreased by 48 %, 56 %, 84 %, and 18 %, respectively (*P* < 0.05). In general, the intake of the WD for 16 weeks induced typical hyperlipidemia, which was compensated by fish oil intake.

### FOH decreased hepatic transaminase activities in WD-fed rats

Plasma ALT and AST levels are important liver injury markers. Compared with the CON group, the ALT and AST levels in rats fed WD were significantly increased (*P* < 0.05), whereas compared with the WD group, the activity of alanine aminotransferase (ALT) and aspartate aminotransferase (AST) in the FOH group was decreased by 17 % (*P* < 0.05) and 28 % (*P* < 0.05), respectively. A similar result was observed in the Glutathione S- transferase (GST) activity. WD feeding increased GST activity compared with CON (*P* < 0.05), but the levels of GST in the FOH and CON groups had no significant difference.

### FOH improved hepatic lipid metabolism in WD-fed rats

The liver-to-body weight ratio of rats in the WD group was 4.79 %, which was significantly higher than that in the CON group, which was 3.09 %, and the ratio in the FOH group was 4.43 %, which was slightly lower than the WD group ( all *P* < 0.05). The hepatic TG and TCh in the WD group were 1.5-fold and 3-fold higher than the CON group, respectively. The rats fed FOH presented 32 % and 64 % decreased hepatic TG and TCh amounts compared with rats fed WD, respectively.

### FOH alleviated liver inflammation in WD-fed rats

**Fig. 1:**
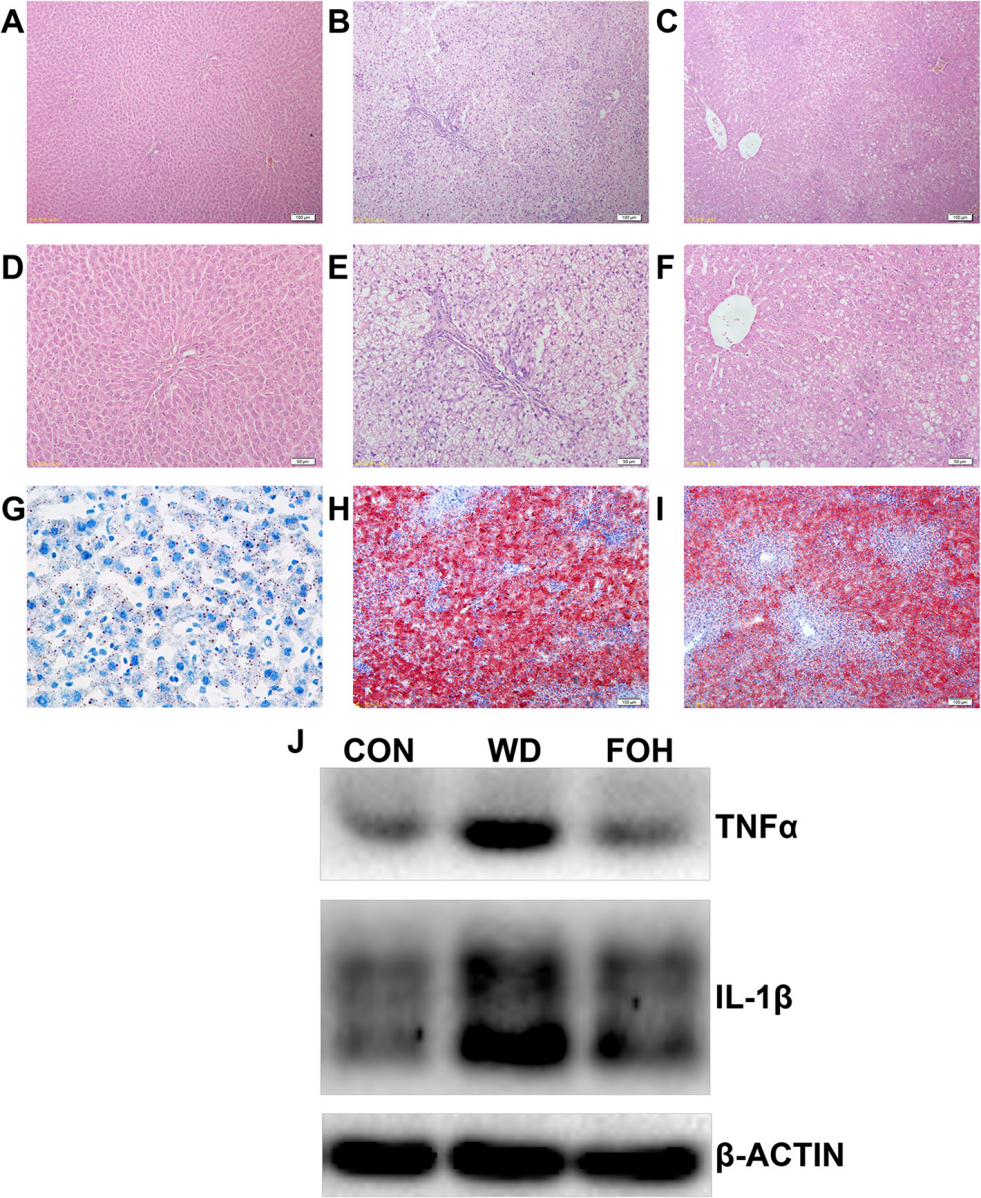
Effects of different diets on rat liver steatosis and inflammation. Male SD rats were fed with normal chow diet, WD, or FOH for 16 weeks, and their liver tissue sections were stained with Hematoxylin and Eosin (H&E, A-F) staining and Oil-red staining(G-I) respectively, and hepatic cytokine expression of TNFα and IL-1β were examined by western blotting (J). The representative photographs as follow: (A-C) H&E staining photomicrographs of the CON, WD and FOH group liver sections (×100), respectively; (D-F) H&E staining photomicrographs of the CON, WD and FOH group liver sections (×200), respecitvely; (G-I) Oil-red staining photomicrographs of the CON, WD and FOH group liver sections (×100), respectively; (J) Differentially expressed level of hepatic TNFα and IL-1β in each groups, β-actin were used as internal control

As shown in Fig. 1 A-F, hematoxylin and eosin (H&E) staining illustrated that WD feeding induced severe macrovesicular steatosis and inflammatory cell infiltration in the liver. FOH dramatically diminished liver steatosis compared with that of WD group. Furthermore, fish oil feeding reduced inflammatory cells infiltration around portal area (Fig. 1A-F). A hepatic histological NAFLD activity scoring system based on vesicular steatosis, hepatocellular ballooning, lobular inflammation, and fibrosis indicated that 16-week WD feeding induced NASH in rats (NAS = 7.83), but the rats in the FOH group only developed simple steatosis without steatohepatitis (NAS = 3.17) (Table 2). Additionally, western blotting analysis indicated that WD feeding up-regulated the hepatic pro-inflammatory cytokines level such as TNF-α and IL-1β, which were rescued by fish oil feeding (Fig. 1J).

**Table 2:** NAFLD activity score (NAS)

	CON	WD	FOH
Steatosis(n)	0(6)	2(1)3(5)	0(2)1(4)
Lobular inflammation(n)	0(6)	2(2)3(4)	1(5)2(1)
Hepatocellular ballooning(n)	0(6)	2(4)3(2)	1(4)2(2)
NAS	0	7.83	3.17
Fibrosis(n)	0(6)	2(6)	1(5)0(1)

NAFLD activity scores (NAS): the unweighted sum of steatosis, lobular inflammation, and hepatocellular ballooning, NAS of >5 correlated with a diagnosis of NASH.

### Differentially expressed genes identified between two groups

**Fig. 2:**
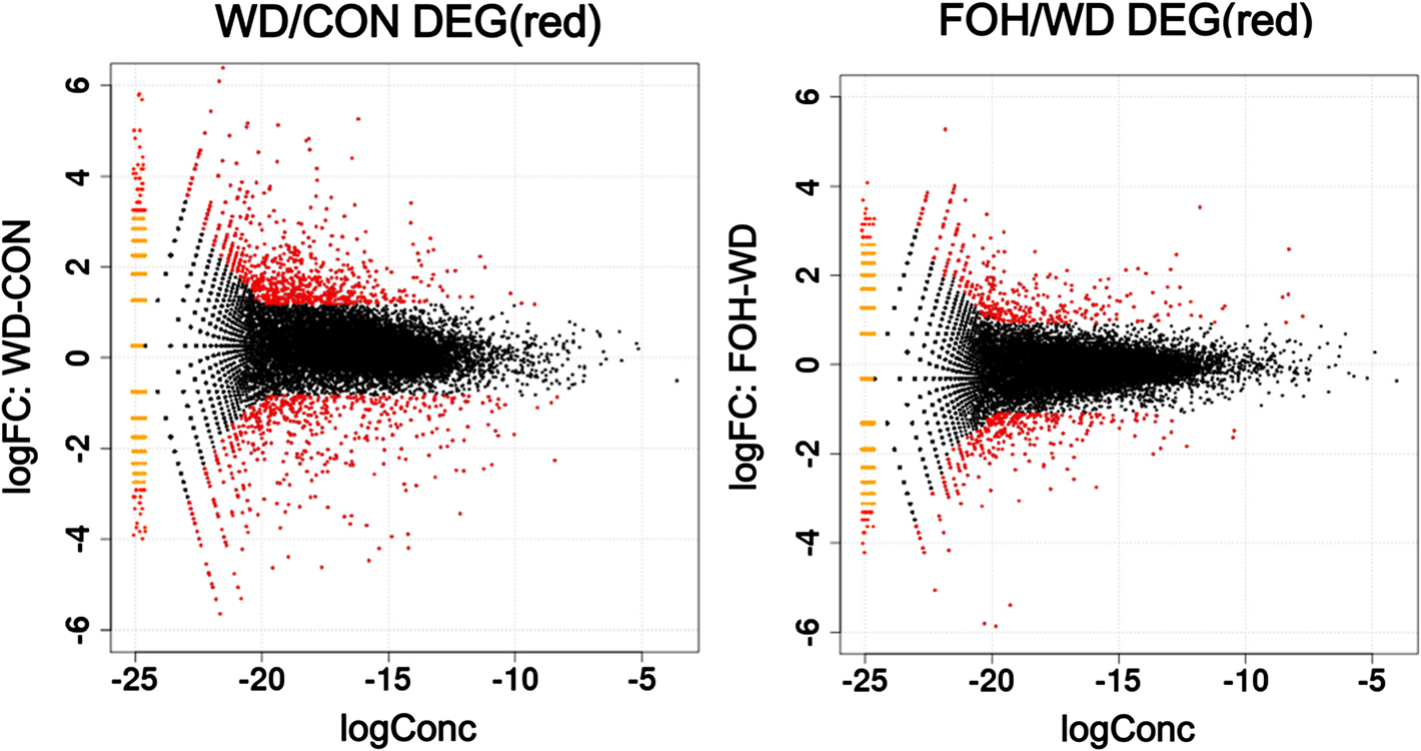
MA plot of differentially expressed genes identified in WD/CON and FOH/WD rat livers. Data represent individual gene responses plotted as log2 fold-change versus base Mean fold-change >2 (*P* < 0.01), with a negative change representing the downregulated genes and a positive change representing the upregulated genes

To analyze differentially expressed genes (DEGs), the genes which were tested with fold change >2 and *P* < 0.01 in the EdgeR package were selected. Compared with the CON group, there were 1379 DEGs found in the WD group, within 827 genes upregulated and 522 downregulated (Additional file 1: Table S4). Compared with the WD group, 711 DEGs were found in the FOH group, among them, the expression of 378 genes was increased and the others were decreased (Additional file 1: Table S5). MA plot analysis showed the magnitude of distribution of significantly changed genes (Fig. 2). Furthermore, via analyzing the overlapped genes between the WD and FOH groups, we found that in the FOH group, the expression of 88 genes which decreased in the WD group were rescued, and the expression of 149 genes increased in the WD group were inhibited.

### Functional enrichment of DEGs in FOH compared with WD

The DAVID platform (data base including Gene ontology, Interpro, and KEGG Gene functions) was adopted for functional cluster. The results demonstrated that in the FOH group, upregulated DEG clusters were mainly involved in fatty acid metabolic process, ion homeostasis, protein phosphorylation, membrane-enclosed lumen, and response to nutrients (Additional file 1: Table S6), while downregulated gene clusters were involved in extracellular matrix, carbohydrate binding, regulation of the system process, response to lipopolysaccharide, and cell adhesion (Additional file 1: Table S7). Some important genes listed and summarized in Tables 3, 4 and 5.

**Table 3:** N-3 PUFA improved hepatic lipid metabolism in FOH group.

Category/Function	Gene symbol / Gene alias	Gene name	Fold change	
			WD*vs.* CON	FOH *vs.* WD
Fatty Acid Transport	Slc27a1/FATP-1	solute carrier family 27 (fatty acid transporter), member1	1.48 ↑	0.85↓
	Slc27a2/ FATP-2	solute carrier family 27 (fatty acid transporter), member 2	1.17 ↑	1.40↑
	Cd36	fatty acid translocase (FAT/CD36)	1.05↓	2.07↑
Fatty Acid Synthesis	Srebf1/SREBP1c	sterol regulatory element binding transcription factor 1	1.72↑	2.27↓
	Fasn	fatty acid synthase	8.54↓	1.99↓
	Acaca	acetyl-CoA carboxylase alpha	3.10↓	1.17↑
	Elovl5	ELOVL fatty acid elongase 5	1.31↓	2.26↓
	Fads1	fatty acid desaturase 1	6.28↓	2.87↓
	Fads2	fatty acid desaturase 2	3.26↓	2.32↓
	Scd1	stearoyl-Coenzyme A desaturase 1	1.49↓	2.64↓
Fatty Acid Degradation	Ppara/PPARα	peroxisome proliferator activated receptor alpha	1.48↓	1.31 ↑
	Cpt1a	carnitine palmitoyltransferase 1a, liver	1.08↑	1.53↑
	Cpt2	carnitine palmitoyltransferase 2	1.01↑	1.42↑
VLDL Secretion	Mttp	microsomal triglyceride transfer protein	1.29↓	1.23↑
	Apob	apolipoprotein B	1.11↓	1.26↑

**Table 4:** N-3 PUFA improved hepatic cholesterol homeostasis in FOH group

Category/Function	Gene symbol / Gene alias	Gene name	Fold change	
			WD*vs.* CON	FOH *vs.* WD
Cholesterol synthesis	Pmvk	phosphomevalonate kinase	2.9 ↓	1.22 ↓
	Fdps	farnesyl diphosphate synthase	6.97 ↓	1.66 ↓
	Nsdhl	NAD(P) dependent steroid dehydrogenase-like	1.95 ↓	1.41 ↓
	Dhcr7	7-dehydrocholesterol reductase	3.13 ↓	1.22 ↓
Cholesterol-rich lipoprotein uptake	Ldlr	LDL receptor	2.56 ↓	1.13 ↑
	Olr1/LOX-1	oxidized low density lipoprotein (lectin-like) receptor 1	60.48 ↑	6.70 ↓
	Scarb1/ SR-BI	Scavenger receptor class B type I (SR-BI)	1.10 ↑	1.37 ↓
	CD36	Cluster differentiation protein 36	1.05 ↓	2.07 ↑
Cholesterol secretion	Abcg1	ATP-binding cassette, subfamily G (WHITE), member 1	3.57 ↑	1.54↑
	Abcg5	ATP-binding cassette, subfamily G (WHITE), member 5	1.48 ↓	1.93↑
	Abcg8	ATP-binding cassette, subfamily G (WHITE), member 8	7.24 ↓	2.7 1↑
Intracellular metabolism to oxysterols and bile acids	Cyp7a1	cytochrome P450, family 7, subfamily a, polypeptide 1	1.53 ↓	1.98↑
	Cyp27a1	cytochrome P450, family 27, subfamily a, polypeptide 1	1.14 ↓	1.08↑

**Table 5:** N-3 PUFA reduced liver inflammation in FOH group

Category/ Function	Gene symbol / Gene alias	Gene name	Fold change	
			WD*vs.*CON	FOH*vs.*WD
CD family	*Cd8a*	CD8 antigen, alpha polypeptide (p32)	2.53 ↑	1.78 ↓
	*Cd44*	CD44 antigen	3.37 ↑	1.66 ↓
	*Cd81/TAPA-1*	CD81 antigen; target of antiproliferative antibody 1	1.17 ↑	1.08 ↓
	*Cd180*	CD180 antigen	1.55 ↑	1.14 ↓
Chemokines and chemokine receptors	*Ccl2/MCP-1*	chemokine (C-C motif) ligand 2; monocyte chemoattractant protein 1	5.73 ↑	2.12 ↓
	*Cxcl1/GROa*	chemokine (C-X-C motif) ligand 1; growth-regulated oncogene, alpha	5.26 ↑	3.92 ↓
	*Cxcl9/MIG*	chemokine (C-X-C motif) ligand 9; monokine induced by interferon-gamma	1.38 ↑	2.10 ↓
	*Cxcl16*	chemokine (C-X-C motif) ligand 16	3.25 ↑	1.66 ↓
	*Cxcr3*	chemokine (C-X-C motif) receptor 3	2.24 ↑	1.29 ↓
	*Cxcr4*	chemokine (C-X-C motif) receptor 4	1.75 ↑	1.58 ↓
Semaphorins, plexins and neuropilins	*Sema4a*	sema domain, immunoglobulin domain (Ig), transmembrane domain (TM) and short cytoplasmic domain, (semaphorin) 4A	1.87 ↑	1.29 ↓
	*Sema4d*	sema domain, immunoglobulin domain (Ig), transmembrane domain (TM) and short cytoplasmic domain, (semaphorin) 4D	2.30 ↑	1.29 ↓
	*Sema6b*	sema domain, transmembrane domain (TM), and cytoplasmic domain, (semaphorin) 6B	2.29 ↑	1.27 ↓
	*Plxnd1*	plexin D1	1.38 ↑	1.10 ↓
JAK-STAT-SOCS pathway	*Jak2*	Janus kinase 2 (a protein tyrosine kinase)	1.23 ↑	1.05 ↓
	*Jak3*	Janus kinase 3 (a protein tyrosine kinase, leukocyte)	1.95 ↑	1.09 ↓
	*Stat3*	signal transducer and activator of transcription 3 (acute-phase response factor)	1.40 ↑	1.33 ↓
	*Stat5b*	signal transducer and activator of transcription 5B	1.48 ↑	1.33 ↓
	*Socs1*	suppressor of cytokine signaling 1	5.09 ↑	7.06 ↓
	*Socs2*	suppressor of cytokine signaling 2	3.46 ↑	1.39 ↓
IFN pathway	*Irf1*	interferon regulatory factor 1	2.07 ↑	1.40 ↓
	*Irf5*	interferon regulatory factor 5	1.71 ↑	1.18 ↓
TNFα and TGFβ pathways	*Ltb*	lymphotoxin beta (TNF superfamily, member 3)	1.99 ↑	2.19 ↓
	*Tnfrsf14*	tumor necrosis factor receptor superfamily, member 14 (herpesvirus entry mediator)	3.99 ↑	1.70 ↓
	*Tnfrsf1b*	tumor necrosis factor receptor superfamily, member 1B	1.56 ↑	1.07 ↓
	*Traf7*	TNF receptor-associated factor 7	1.94 ↑	1.67 ↓
	*Tgfb1*	transforming growth factor, beta 1	2.31 ↑	1.02 ↓
	*Tgfbr2*	transforming growth factor, beta receptor II (70/80 kDa)	1.30 ↑	1.21 ↓
NFκB pathway	*Ikbkb*	inhibitor of kappa light polypeptide gene enhancer in B-cells, kinase beta	1.21 ↑	1.05 ↓
	*Nfkb1*	nuclear factor of kappa light polypeptide gene enhancer in B-cells 1 (p105)	1.45 ↑	1.19 ↓
	*Nfkb2*	nuclear factor of kappa light polypeptide gene enhancer in B-cells 2 (p49/p100)	1.95 ↑	1.33 ↓
Toll-like receptors and lipopolysaccharide signaling pathway	*Tlr2*	toll-like receptor 2	1.86 ↑	1.02 ↓
	*Cd14*	CD14 antigen (lipopolysaccharide receptor)	1.50 ↑	1.16 ↓
	*Litaf*	lipopolysaccharide-induced TNF factor	2.47 ↑	1.54 ↓
Matrix proteases	*Mmp9*	matrix metallopeptidase 9 (gelatinase B, 92 kDa gelatinase, 92 kDa type IV collagenase)	6.03 ↑	2.05 ↓
	*Mmp14*	matrix metallopeptidase 14 (membrane-inserted)	1.30 ↑	1.02 ↓
	*Plat/tPA*	plasminogen activator, tissue	2.15 ↑	1.53 ↓
	*Plau/uPA*	plasminogen activator, urokinase	1.50 ↑	1.66 ↓

### Validation of RNA-seq data by quantitative RT-PCR

**Fig. 3:**
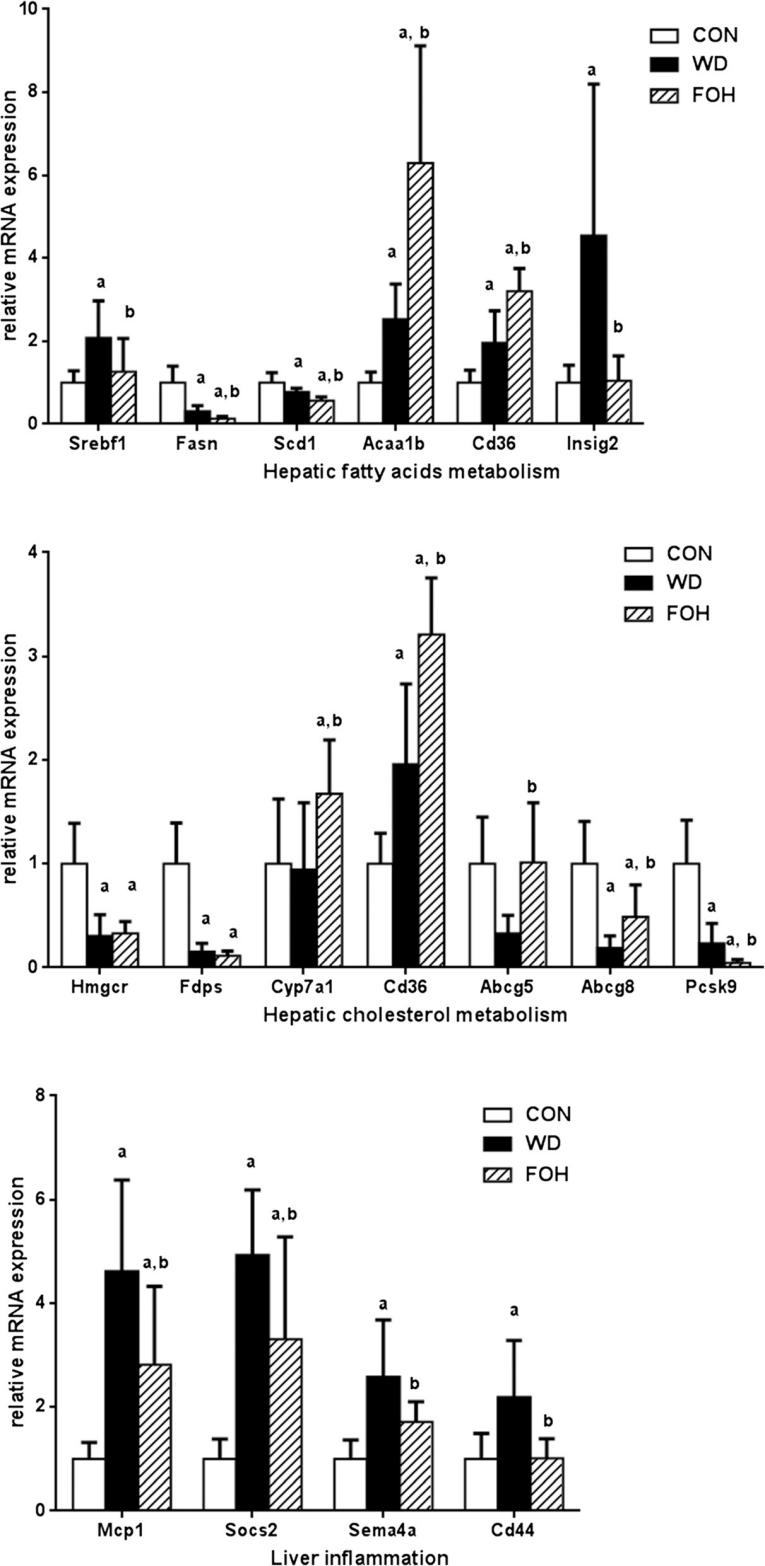
qRT-PCR results of selected genes. The mRNA expression levels of selected genes involved in hepatic fatty acid (A), cholesterol (B) metabolism and liver inflammation (C) were examined by qRT-PCR. Values are expressed as mean ± SD; n = 10 in each treatment group. ^a^ *P* < 0.05 vs. CON group; ^b^ *P* < 0.05 vs. WD group.

**Fig. 4:**
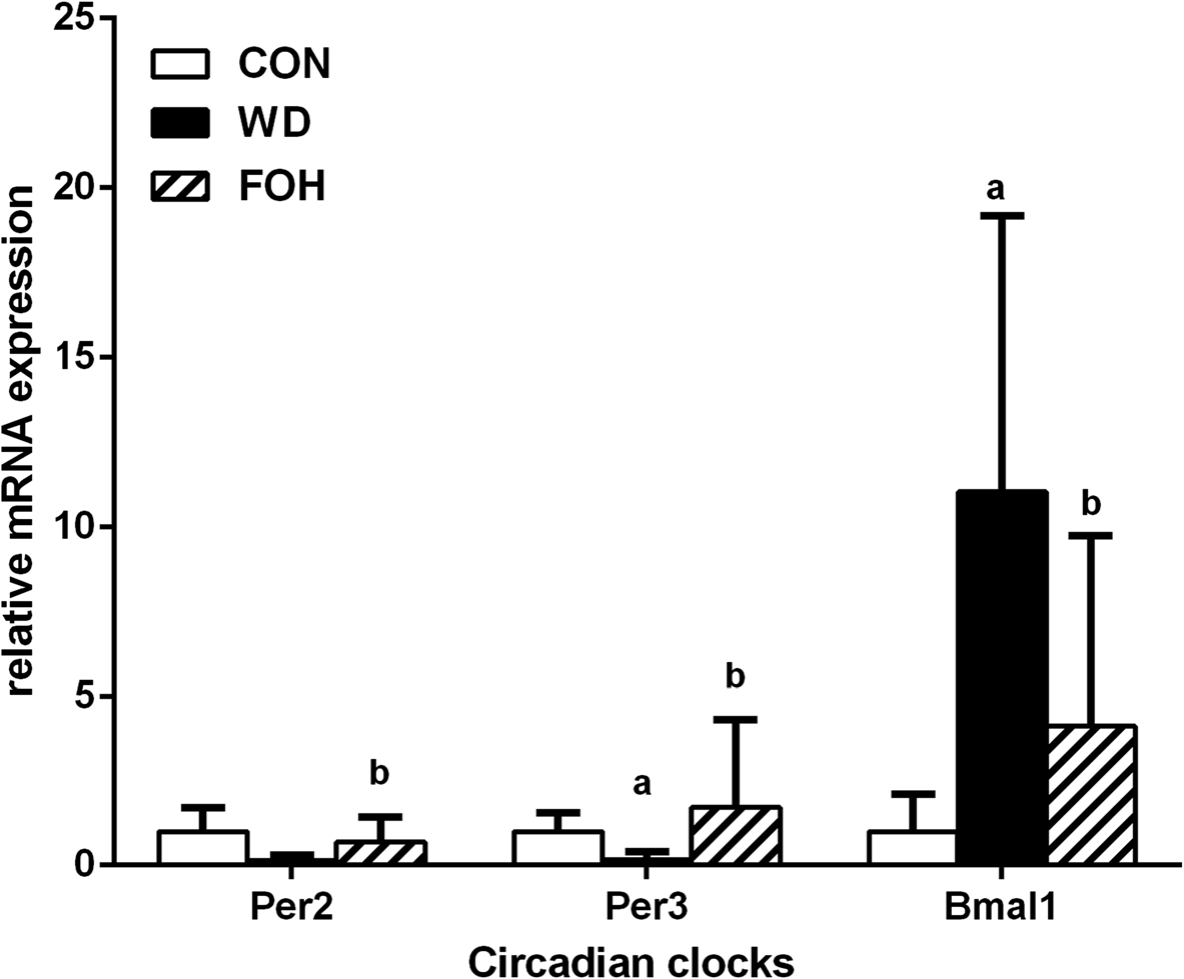
Effects of diets on *Per2*,*Per3* and *Bmal1* expression. The mRNA expression levels of *per2, per3* and *Bmal1* were examined by qRT-PCR. Values are expressed as mean ± SD; n = 10 in each treatment group. a: vs CON group; b: vs WD group, *P* < 0.05

To verify the RNA-seq data, the expression of selected genes involved in lipid metabolism and inflammatory immune response were examined by quantitative real-time RT-PCR (qRT-PCR). The WD feeding significantly increased the expression of sterol regulatory element binding factor 1 (*Srebf1*), suppressor of cytokine signaling 2 (*Socs2*), monocyte chemoattractant protein 1 (*Mcp1*) and semaphorin 4A (*Sema4a*), and brain and muscle ARNT-like protein 1 (*Bmal1*) whereas the regulatory effects of WD were significantly reversed by intake of fish oil (Figs. 3 and 4). Moreover, WD feeding downregulated the expression of fatty acid synthase (*Fasn*) and stearoyl-Coenzyme A desaturase 1 (*Scd1*), whereas the expression in the FOH group was further decreased (Fig. 3). Additionally, WD feeding decreased the expression level of period gene 2 and 3 (*Per2* and *Per3*) , and the effects were rescued by fish oil feeding (Fig. 4). The results of qPCR were consistent with our RNA-seq data.

## Discussion

This study was focused on the protective effects of n-3 PUFA-rich (e.g. DHA and EPA) fish oil against WD-induced NAFLD. To get a better observation of the protective effects of fish oil, we fed WD animals with high-dose fish oil (10 % [w/w] in diet) for 16 weeks. In this study, we applied a next-generation high-throughput sequencing technology to investigate the difference of hepatic mRNA expression profile between WD-fed NAFLD rats and FOH-fed rats. Our findings demonstrated that fish oil consumption rescued the effects of WD on the hepatic fatty acids and cholesterol accumulation, metaflammation and rhythm disturbance, suggesting that high dose fish oil supplement may be useful for clinical NAFLD treatment.

### n-3 PUFA improved hepatic lipid metabolism

TG ectopic accumulation in liver plays a central role in the development of NAFLD. In the present study, the plasma lipid analysis and Oil red staining showed that 16 weeks of fish oil feeding significantly reduced plasma TG and hepatic steatosis compared with the WD group. Additionally, our transcriptomic study compared the hepatic mRNA expression level between FOH group and WD group, and Gene Oncology (GO) annotation enriched a number of DEGs connected to fatty acids metabolism (Table 3, 4). More than one fourth of liver TG are synthesized *de novo* [17]. SREBP1c, encoded by the gene *Srebf1*, plays a crucial role in *de novo* lipogenesis. Our transcriptomic results demonstrated that the WD raised the expression of *Srebf1,* while the fish oil feeding abrogated this effect and the *Srebf1* expression was normalized to the level similar to the control diet group. Meanwhile, the mRNA levels of SREBP1c downstream genes involved in *de novo* lipogenesis, such as *Elovl5, Fads1/2* and *Scd1* in FOH group were dramatically lower than the WD group (Additional file 1: Table S4, S5, and Fig. 3). Several studies also indicated the inhibitory effects of fish oil on high-fat diet-induced SREBP1c overexpression [18, 19]. The suppressive effects of PUFAs on SREBP1c was occurred at post-transcriptional level [20]. Interestingly, the expression level of *insig2* in FOH group is similar to that in CON group and significantly lower than that in WD group (Additional file 1: Table S4, S5). This finding was consistent with the effects of DHA on hepatocyte *in vitro* [21]. When animal insulin levels are high, INSIG2 improves SREBP1c cleavage and mature in Golgi, and Insig2 itself undergoes ubiquitin related proteasomal degradation [22, 23]. The differentially expressed *Srebf1* and *Insig2* between CON, WD and FOH groups suggest that high fat feeding induced *de novo* lipogenesis mainly attributed to highly S*rebf1* expression in rats but not hyperinsulinemia induced SREBP1c mature, and the process was rescued by fish oil feeding. Consistently, neither high-fat WD nor fish oil feeding showed the effects on fasting plasma glucose, and other studies also pointed out that rats are more tolerant to high-fat-diet–induced insulin resistance [24].

Besides the *de novo* lipogenesis, the expression of genes involved in liver lipid uptake from the circulation (CD36 and slc27a2) [17], fatty acids beta-oxidation in the mitochondria (*Cpt1a, Cpt2*, *Acadl,* and *Acaa1b*) and TG export (*Apob* and *Mttp*) [25, 26] were regulated by fish oil feeding. These findings imply that fish oil can promote hepatic fatty acids beta-oxidation and TG excretion, contribute to the amelioration of liver steatosis (Fig. 1, Table 3).

Beside the hepatic triglycerides metabolism, fish oil rich in n-3 PUFAs improved hepatic cholesterol metabolism (Table 4). In the present study, 16-week WD (2 % cholesterol, w/w) feeding induced hypercholesterolemia and hepatic cholesterol overload in SD rats, and fish oil feeding significantly reduced the cholesterol content in blood, particularly in the form of LDL-C (Table 1). In the transcriptomic results, a series of DEGs between CON *vs.* WD or FOH *vs.* WD group were enriched in cholesterol metabolism pathways. Though both high caloric diets intake decreased the expression of key rate-limiting enzyme in cholesterol synthesis: 3-hydroxy-3-methylglutaryl- coenzyme A reductase (HMGCoAR) in the liver, fish oil consumption regulated the hepatic expression of a serial of genes related to cholesterol transport, including: *Fat/CD36*, Lox1 and *Scarb1* [27, 28], which were associated with cholesterol uptake; *Abcg5/8*, which were involved in cholesterol excretion [29]; and *Cyp7a1* and *Cyp27a1*, which were connected to bile acid excretion [30].

Interestingly, our transcriptomic data indicated that WD feeding reduced the expression of *pcsk9* and *ldlr* compared with CON group, while the fish oil consumption furtherly inhibited the expression of *pcsk9* (Fig. 3). Proprotein convertase subtilisin/kexin type 9 (PCSK9) plays an important role in degradation of LDLR, and it is thought a valuable treatment target of hypercholesterolemia [31, 32]. A recent clinical study has indicated that fish oil supplement could reduce plasma PCSK9 level in women [33]. Our finding suggests that fish oil may protect against WD-induced hypercholesterolemia via reducing the hepatic *psck9* expression. The effects of fish oil on PCSK9 is worthy further study.

### Circadian clocks modification may contribute to the protective effects of fish oil on hepatic lipid metabolism

Our study confirmed the previous findings that n-3 PUFAs can ameliorate high-fat-diet–induced hepatic lipid accumulation [34]. However, how fish oil protects the liver against lipid and cholesterol metabolism disorder is still unclear. Circadian clock plays a crucial role in metabolism and energy regulation, and the GO enrichment analysis presented several DEGs between FOH and WD group were associated with circadian clock modification. Period genes (*Pers*) are in charge of negative feedback loop of circadian clock control, as well as the *Bmal1* plays the central role in circadian gene transcription regulation [35]. In our study, the expression of both *per2* and *per3* were decreased in the WD group, and the expression of *Bmal1* was increased, which may lead to disruption of circadian clock and metabolic disorder, and the disturbed expression of *per2, per3* and *Bmal1* were all rescued by fish oil feeding (Fig. 4). Our RNA-seq data suggests that fish oil may protect the liver against NAFLD via modification of the circadian clocks.

### n-3 PUFA ameliorated hepatic inflammation

High fat diet induced chronic low-grade inflammation in liver, known as metaflammation [36]. The histological staining and western blotting analysis illustrated that the fish oil intake partly alleviated WD-induced inflammatory cells infiltration pro-inflammatory cytokines expression in liver (Fig. 1 A-F, J). The transcriptomic analysis enriched a set of DEGs involved in inflammation such as chemokine synthesis, bacteria response, and extracellular matrix accumulation were upregulated, whereas n-3 PUFAs consumption reduced the expression of genes related to CD, chemokines and their receptors, semaphorins and their receptors, JAK-STAT-SOCS pathway, interferon pathway, NFκB pathways, and TLR pathways (Table 5). Similar to the findings of Ito *et al*., WD intake raised the expression of *Ccl2*, which plays an important role in hepatic steatosis and inflammation [37]. The expression of *Ccl2* was reduced in the FOH group compared with the WD group, suggesting that the modification of *Ccl2* expression might contribute to the hepatoprotective effects of n-3 PUFA. Egan et al. showed that *Cd44*, an adhesion molecule in charge of cell migration, is associated with the leukocyte recruitment in a lithogenic diet-induced hepatic steatosis[38]. In the present study, the expression of *Cd44* in the FOH group was lower than WD group, implying that n-3 PUFAs reduce the hepatic inflammatory cell infiltration in WD-induced NAFLD. Bertola et.al [39] found that the expression of inflammatory factors in plexin/semaphorin family was significantly increased in NASH patients, and in this study, n-3 PUFA feeding restored WD-induced expression of T-helper 1 leukocyte differentiation-related *Sema4a* and T-leukocyte activation-related *Sema4d*. We found that the expression of *Socs2*, a key molecule in JAK-STAT-SOC pathway, was elevated in the WD group, and the expression in FOH group was lower than the WD group, which is consistent with the results of Zadjali et.al. [40], suggesting that n-3 PUFA might protect against diet-induced NAFLD via JAK-STAT-SOC pathway. These findings suggests that fish oil protect liver against WD-induced metaflammation mainly via suppressing the infiltration and activation of hepatic lymphocytes.

## Conclusions

The present study demonstrated that chronic WD intake induced hepatic TG accumulation, hepatocellular lipid and cholesterol metabolic chaos, metaflammation and fibrogenesis in the progress of NAFLD. The n-3 PUFA intake compensated for WD-induced steatosis and depressed hepatic inflammation, which contribute to the amelioration of diet-induced NAFLD progress. The results suggest that n-3 PUFAs might be a potential dietary therapeutic tool against NAFLD.

## Methods

### Animals

Male Sprague–Dawley rats (8–9 weeks old weighing 180 ~ 200 g) were purchased from Tonji Medical College of Huazhong University of Science and Technology (Wuhan, China). They were housed five per cage and maintained in a 12-hour day/night cycle and kept under standard laboratory conditions with a room temperature of 22 °C ± 1 °C, relative humidity 60 % ±10 %, and 20 air changes per hour. The rats were acclimatized to laboratory conditions for 1 week before the experiment and randomly allocated to three groups of 10 animals each fed for 16 weeks: normal chow diet[41] with 10 kcal% fat (CON group), Western style lard-rich diet with 45 kcal% fat and 2 % cholesterol (w/w) (WD group), or fish-oil-rich diet with 10 % fish oil (w/w) and a total 45 kcal% fat with 2 % cholesterol (w/w) (FOH group). Pure fish oil (Coland, China) was obtained from anchovy. The animals were given water and diet ad libitum. This animal study was conducted according to the Guidelines for the Care and Use of Experimental Animals, and the protocol was approved by Laboratory Animal Ethics Committee of Wuhan Polytechnic University (ID Number: 20121009006).

The diets followed AIN93’s recommendations. Additional file 1: Table S1 lists the nutrient composition, energy ratio, and fatty acid compositions in different feedstuff. At the end of 16th week of the study, 12-hour fasted rats were sacrificed with CO_2_ suffocate[42]. Blood was collected into a heparinized tube (6–8 mL) and centrifuged at 1000 × *g* for 15 min at 4 °C, and plasma was collected and stored at −80 °C until analysis. The liver was quickly removed, rinsed with 0.9 % sodium chloride solution and weighed; a portion of the right hepatic lobe was either frozen in liquid nitrogen and kept at −80 °C, or fixed in 10 % neutral-buffered formalin and embedded in paraffin for histological studies.

Plasma glucose, triacylglycerol (TG), total cholesterol (Tch), high-density lipoprotein cholesterol (HDL-C), and low-density lipoprotein cholesterol (LDL-C) concentrations were measured using spectrophotometric clinical assays, and enzymatic activities of plasma alanine aminotransferase (ALT) and aspartate aminotransferase (AST) were tested according to the protocol. All assays kits were bought from Nanjing Jiancheng Institute of Biological Engineering (Nankin, China). All tests were conducted according to the standard process by spectrophotometry (Beckman, Inc., USA).

### Histological studies

Fresh liver samples from the right lobe were either frozen and stained with Oil Red O or fixed in 10 % neutral-buffered formalin and embedded in paraffin and sectioned at 5-μm thickness, stained with hematoxylin and eosin (H&E). Histological steatosis and inflammation were assessed semi-quantitatively by a pathologist who was blinded to the experimental protocol, according to the scoring system proposed by Kleiner et al.[43].

### Western blot analysis

Total protein extraction from rat livers was performed according to a commercially available kit (23252; Pierce™ Microplate BCA Protein Assay Kit, USA). Equal amounts of protein (40–80 μg) were separated by 10 % SDS-PAGE and electro-transferred to a PVDF membrane (Millipore). Membranes were blocked, and then incubated with antibodies overnight, such as, anti-IL-1β (1:500, H-153; sc-7884; Santa Cruz), anti-TNFα (1:500, L-19; sc-1351; Santa Cruz), anti-β-actin (1:500, C4; sc-47778; Santa Cruz), and then with the horseradish peroxidase-conjugated secondary antibody for 2 h. Specific protein expression levels were normalized to β-actin for total protein analyses. The blot was detected with the ChemiDoc™ MP System detection system (Bio-Rad Laboratories, Inc). The experiments were replicated three times. The membranes were scanned and the sum optical density was quantitatively analyzed by Image Lab software (Bio-Rad Laboratories, Inc).

### RNA isolation, library preparation, and sequencing

Total RNA isolated from livers (liver tissues of ten rats per group were pooled together) was treated with RQ1 DNase (Promega, Madison, WI) to remove DNA. The pooled sample for each group was prepared using an equal amount of total RNA from each individual . Polyadenylated mRNAs were purified and concentrated with oligo(dT)-conjugated magnetic beads (Invitrogen, Carlsbad, CA) before use for directional RNA-seq library preparation. Purified mRNAs were iron-fragmented at 95 °C followed by end repair and 5' adaptor ligation, followed by reverse transcription with RT primer harboring 3' adaptor sequence and randomized hexamer. The cDNAs were purified and amplified and PCR products corresponding to 200–500 bps were purified, quantified, and stored at −80 °C until used for sequencing. For high-throughput sequencing, the libraries were prepared following the manufacturer’s instructions and applied to Hiseq 2000 system for 100 nt pair-end sequencing by ABlife (Wuhan, China).

### RNA-seq raw data clean and alignment statistics

Raw reads were first discarded if they contained more than 2-N bases, and the reads were processed by clipping adaptor and removing low-quality bases. Very short reads (less than 20 nt) were also dropped. FASTX-Toolkit (Version 0.0.13) was used to obtain clean reads. The clean reads were aligned to the reference genome by bowtie[44] or Tophat[45]. Based on the gene annotation of the genome, the aligned reads with more than one genome location were discarded due to their ambiguous location. Uniquely localized reads were used to calculate the number of reads and RPKM value (RPKM represents reads per kilobase and per million) for each gene, according to the genome location of reads and genes. Other statistical results, such as gene coverage and depth, distribution of reads around start and stop codons, were also obtained.

### Differentially expressed gene analysis

DEGs between the test and control samples were analyzed using edgeR [46], one of the R packages. For each gene, the p-value was computed and the significance threshold to control FDR at a given value was calculated. The fold-changes were also estimated within the edgeR statistical package.

### Functional enrichment analysis

The lists of differentially expressed genes were submitted to DAVID web server (https://david.ncifcrf.gov/) for enrichment analysis. The enrichment clusters were sorted by the enrichment score on descending order. The categories of one cluster were sorted by P-value in descending order. Fold Enrichment, Bonferroni and Benjamini corrected P-value and FDR were also presented for each category of each cluster.

### Validation of DEGs by qRT-PCR

To evaluate the repeatability and reproducibility of gene expression data obtained by RNA-Seq, an qRT-PCR assay using SYBR Green chemistry (SYBR® qPCR Mix, Rox; TAKARA Biotechnology, Dalian, China). The isolated RNA of individual samples was reverse-transcribed into cDNA using high-capacity reverse cDNA transcription kit (Applied Biosystems, Foster City, CA) in a total volume of 20 μL containing 1 μg of total RNA, following the manufacturer’s instructions. PCR primers were designed using Primer Express software (Applied Biosystems). Additional file 1: Table S2 shows the PCR primers sequences. Rat GAPDH was simultaneously used as endogenous control gene. Each SYBR green-based real-time PCR reaction contains, in a final volume of 14 μl, cDNA from 14 ng of reverse-transcribed total RNA, 2.33 pmol primers, and 7 μl of 2× SYBR Green PCR Master Mix (Applied Biosystems). Triplicate PCR reactions were carried out in 96-well plates using 7500 Real-Time PCR System (Applied Biosystems). The conditions were 50 °C for 2 min, 95 °C for 10 min, followed by 40 cycles of 95 °C for 15 s and 60 °C for 1 min. The gene expression level was normalized to that of invariable control gene, GAPDH. Data are presented as minus Δ cycle threshold Ct.

### Statistical analysis

Data are presented as mean ± SD. One-way ANOVA and Tukey’s honestly significant difference post hoc analysis were conducted to establish significant differences. All analyses were performed by SPSS 19.0 statistical software. Differences were considered statistically significant at *P <* 0.05.

## Additional file

Additional file 1:
**Table S1.** The nutrient composition, energy ratio and fatty acid profiles of experimental diets. **Table S2**. Primers used in this study. **Figure S3**. The weekly body weight change of experiment animals. **Table S4**. Differentially expressed genes between WD group and CON group. **Table S5**. Differentially expressed genes between FOH group and WD group. **Table S6**. Upregulated DEGs (FOH vs. WD) implicated in the enrichedgene ontology processes. **Table S7**. Downregulated DEGs (FOH vs. WD) implicated in the enrichedgene ontology processes. (ZIP 236 kb)

## Abbreviations

PUFAs: n-3 polyunsaturated fatty acids
*NAFLD*: Non-alcoholic fatty liver disease
CON: Control diet
WD: A Western style high-fat and high-cholesterol diet
FOH: A WD diet combined with fish oil
RT-PCR: Reverse transcription-polymerase chain reaction
TG: Triacylglycerol
NASH: Non-alcoholic steatohepatitis
ERS: Endoplasmic reticulum stress
FFAs: Free fatty acids
TLR: Toll-like receptor
DHA: Docosahexaenoic acid
NEFA: Non-esterified fatty acid
Tch: Total cholesterol
HDL-C: High-density lipoprotein cholesterol
LDL-C: Low-density lipoprotein cholesterol
ALT: Alanine aminotransferase
AST: Aspartate aminotransferase
GST: Glutathione S- transferase
H&E: Hematoxylin and eosin
DEGs: Differentially expressed genes
Srebf1: Sterol regulatory element binding factor 1
*Socs2*: Suppressor of cytokine signaling 2
*Mcp1*: Monocyte chemoattractant protein 1
Ig: Sema domain, immunoglobulin domain
TM: Transmembrane domain
*Sema4a*: Semaphorin 4A
*Fasn*: Fatty acid synthase
*Scd1*: Stearoyl-Coenzyme A desaturase 1
VLDL: Very low-density lipoprotein
HMGCoAR: 3-hydroxy-3-methylglutaryl-Coenzymereductase
*Pers*: Period genes

## References

[1] Lonardo A, Ballestri S, Marchesini G, Angulo P, Loria P (2015). Nonalcoholic fatty liver disease: a precursor of the metabolic syndrome. Dig Liver Dis.

[2] Maurantonio M, Ballestri S, Odoardi MR, Lonardo A, Loria P (2011). Treatment of atherogenic liver based on the pathogenesis of nonalcoholic fatty liver disease: a novel approach to reduce cardiovascular risk?. Arch Med Res.

[3] Lonardo A, Sookoian S, Pirola CJ, Targher G: Non-alcoholic fatty liver disease and risk of cardiovascular disease. Metabolism 2015. 10.1016/j.metabol.2015.09.017 26477269

[4] McCullough AJ (2006). Pathophysiology of nonalcoholic steatohepatitis. J Clin Gastroenterol.

[5] Nascimbeni F, Pais R, Bellentani S, Day CP, Ratziu V, Loria P (2013). From NAFLD in clinical practice to answers from guidelines. J Hepatol.

[6] Lonardo A, Bellentani S, Argo CK, Ballestri S, Byrne CD, non-alcoholic fatty liver disease study group dttmoPPL (2015). Epidemiological modifiers of non-alcoholic fatty liver disease: Focus on high-risk groups. Dig Liver Dis.

[7] Rosso N, Chavez-Tapia NC, Tiribelli C, Bellentani S (2014). Translational approaches: from fatty liver to non-alcoholic steatohepatitis. World J Gastroenterol.

[8] Tilg H, Moschen AR (2010). Evolution of inflammation in nonalcoholic fatty liver disease: the multiple parallel hits hypothesis. Hepatology.

[9] Day CP, James OF (1998). Steatohepatitis: a tale of two "hits"?. Gastroenterology.

[10] Te Sligte K, Bourass I, Sels JP, Driessen A, Stockbrugger RW, Koek GH (2004). Non-alcoholic steatohepatitis: review of a growing medical problem. Eur J Intern Med.

[11] Musso G, Gambino R, Pacini G, De Michieli F, Cassader M (2009). Prolonged saturated fat-induced, glucose-dependent insulinotropic polypeptide elevation is associated with adipokine imbalance and liver injury in nonalcoholic steatohepatitis: dysregulated enteroadipocyte axis as a novel feature of fatty liver. Am J Clin Nutr.

[12] Tomita K, Teratani T, Yokoyama H, Suzuki T, Irie R, Ebinuma H (2011). Plasma free myristic acid proportion is a predictor of nonalcoholic steatohepatitis. Dig Dis Sci.

[13] Lomonaco R, Ortiz-Lopez C, Orsak B, Finch J, Webb A, Bril F (2011). Role of ethnicity in overweight and obese patients with nonalcoholic steatohepatitis. Hepatology.

[14] Musso G, Gambino R, De Michieli F, Cassader M, Rizzetto M, Durazzo M (2003). Dietary habits and their relations to insulin resistance and postprandial lipemia in nonalcoholic steatohepatitis. Hepatology.

[15] Gentile CL, Pagliassotti MJ (2008). The role of fatty acids in the development and progression of nonalcoholic fatty liver disease. J Nutr Biochem.

[16] Parker HM, Johnson NA, Burdon CA, Cohn JS, O'Connor HT, George J (2012). Omega-3 supplementation and non-alcoholic fatty liver disease: a systematic review and meta-analysis. J Hepatol.

[17] Kawano Y, Cohen DE (2013). Mechanisms of hepatic triglyceride accumulation in non-alcoholic fatty liver disease. J Gastroenterol.

[18] Ou J, Tu H, Shan B, Luk A, DeBose-Boyd RA, Bashmakov Y (2001). Unsaturated fatty acids inhibit transcription of the sterol regulatory element-binding protein-1c (SREBP-1c) gene by antagonizing ligand-dependent activation of the LXR. Proc Natl Acad Sci U S A.

[19] Levy JR, Clore JN, Stevens W (2004). Dietary n-3 polyunsaturated fatty acids decrease hepatic triglycerides in Fischer 344 rats. Hepatology.

[20] Xu J, Nakamura MT, Cho HP, Clarke SD (1999). Sterol regulatory element binding protein-1 expression is suppressed by dietary polyunsaturated fatty acids. A mechanism for the coordinate suppression of lipogenic genes by polyunsaturated fats. J Biol Chem.

[21] Botolin D, Wang Y, Christian B, Jump DB (2006). Docosahexaneoic acid (22:6, n-3) regulates rat hepatocyte SREBP-1 nuclear abundance by Erk- and 26S proteasome-dependent pathways. J Lipid Res.

[22] Raghow R, Yellaturu C, Deng X, Park EA, Elam MB (2008). SREBPs: the crossroads of physiological and pathological lipid homeostasis. Trends Endocrinol Metab.

[23] Yellaturu CR, Deng X, Park EA, Raghow R, Elam MB (2009). Insulin enhances the biogenesis of nuclear sterol regulatory element-binding protein (SREBP)-1c by posttranscriptional down-regulation of Insig-2A and its dissociation from SREBP cleavage-activating protein (SCAP).SREBP-1c complex. J Biol Chem.

[24] Romestaing C, Piquet MA, Bedu E, Rouleau V, Dautresme M, Hourmand-Ollivier I (2007). Long term highly saturated fat diet does not induce NASH in Wistar rats. Nutr Metab (Lond).

[25] Kerner J, Hoppel C (2000). Fatty acid import into mitochondria. Biochimica et Biophysica Acta (BBA).

[26] Donnelly KL, Smith CI, Schwarzenberg SJ, Jessurun J, Boldt MD, Parks EJ (2005). Sources of fatty acids stored in liver and secreted via lipoproteins in patients with nonalcoholic fatty liver disease. J Clin Invest.

[27] Musso G, Gambino R, Cassader M (2013). Cholesterol metabolism and the pathogenesis of non-alcoholic steatohepatitis. Prog Lipid Res.

[28] Hui DY, Labonte ED, Howles PN (2008). Development and physiological regulation of intestinal lipid absorption. III. Intestinal transporters and cholesterol absorption. Am J Physiol Gastrointest Liver Physiol.

[29] Baldán Á, Bojanic DD, Edwards PA (2009). The ABCs of sterol transport. J Lipid Res.

[30] Langston TB, Hylemon PB, Grogan WM (2005). Over-expression of hepatic neutral cytosolic cholesteryl ester hydrolase in mice increases free cholesterol and reduces expression of HMG-CoAR, CYP27, and CYP7A1. Lipids.

[31] Lagace TA (2014). PCSK9 and LDLR degradation: regulatory mechanisms in circulation and in cells. Curr Opin Lipidol.

[32] Cohen J, Pertsemlidis A, Kotowski IK, Graham R, Garcia CK, Hobbs HH (2005). Low LDL cholesterol in individuals of African descent resulting from frequent nonsense mutations in PCSK9. Nat Genet.

[33] Graversen CB, Lundbye-Christensen S, Thomsen B, Christensen JH, Schmidt EB (2015). Marine n-3 polyunsaturated fatty acids lower plasma proprotein convertase subtilisin kexin type 9 levels in pre- and postmenopausal women: A randomised study. Vascul Pharmacol.

[34] Shapiro H, Tehilla M, Attal-Singer J, Bruck R, Luzzatti R, Singer P (2011). The therapeutic potential of long-chain omega-3 fatty acids in nonalcoholic fatty liver disease. Clin Nutr.

[35] Marcheva B, Ramsey KM, Buhr ED, Kobayashi Y, Su H, Ko CH (2010). Disruption of the clock components CLOCK and BMAL1 leads to hypoinsulinaemia and diabetes. Nature.

[36] Gregor MF, Hotamisligil GS (2011). Inflammatory Mechanisms in Obesity. Annu Rev Immunol.

[37] Ito M, Suzuki J, Tsujioka S, Sasaki M, Gomori A, Shirakura T (2007). Longitudinal analysis of murine steatohepatitis model induced by chronic exposure to high-fat diet. Hepatol Res.

[38] Egan CE, Daugherity EK, Rogers AB, Abi Abdallah DS, Denkers EY, Maurer KJ (2013). CCR2 and CD44 promote inflammatory cell recruitment during fatty liver formation in a lithogenic diet fed mouse model. PLoS One.

[39] Bertola A, Bonnafous S, Anty R, Patouraux S, Saint-Paul MC, Iannelli A (2010). Hepatic expression patterns of inflammatory and immune response genes associated with obesity and NASH in morbidly obese patients. PLoS One.

[40] Zadjali F, Santana-Farre R, Vesterlund M, Carow B, Mirecki-Garrido M, Hernandez-Hernandez I (2012). SOCS2 deletion protects against hepatic steatosis but worsens insulin resistance in high-fat-diet-fed mice. FASEB J.

[41] Reeves PG, Nielsen FH, Fahey GC (1993). AIN-93 purified diets for laboratory rodents: final report of the American Institute of Nutrition ad hoc writing committee on the reformulation of the AIN-76A rodent diet. J Nutr.

[42] Lin HY, Chen CC, Chen YJ, Lin YY, Mersmann HJ, Ding ST (2014). Enhanced amelioration of high-fat diet-induced fatty liver by docosahexaenoic acid and lysine supplementations. Biomed Res Int.

[43] Kleiner DE, Brunt EM, Van Natta M, Behling C, Contos MJ, Cummings OW (2005). Design and validation of a histological scoring system for nonalcoholic fatty liver disease. Hepatology.

[44] Langmead B, Trapnell C, Pop M, Salzberg SL (2009). Ultrafast and memory-efficient alignment of short DNA sequences to the human genome. Genome Biol.

[45] Trapnell C, Pachter L, Salzberg SL (2009). TopHat: discovering splice junctions with RNA-Seq. Bioinformatics.

[46] Robinson MD, McCarthy DJ, Smyth GK (2010). edgeR: a Bioconductor package for differential expression analysis of digital gene expression data. Bioinformatics.

