# Supplementary material for: Fish oil alleviated high-fat diet–induced non-alcoholic fatty liver disease via regulating hepatic lipids metabolism and metaflammation: a transcriptomic study
Source: Lipids Health Dis. 2016 Feb 1;15:20. doi: 10.1186/s12944-016-0190-y (PMC4736290; doi:10.1186/s12944-016-0190-y)
Supplement: Additional file 1: — Table S1. The nutrient composition, energy ratio and fatty acid profiles of experimental diets. Table S2. Primers used in this study. Figure S3. The weekly body weight change of experiment animals. Table S4. Differentially expressed genes between WD group and CON group. Table S5. Differentially expressed genes between FOH group and WD group. Table S6. Upregulated DEGs (FOH vs. WD) implicated in the enrichedgene ontology processes. Table S7. Downregulated DEGs (FOH vs. WD) implicated in the enrichedgene ontology processes. (ZIP 236 kb) [file 12944_2016_190_MOESM1_ESM.zip › supplements.doc]

**Table S1. Nutrient composition, energy ratio and fatty acids profile of experimental diets.**

|  | 10% fat diet | 45% fat diets | |
| --- | --- | --- | --- |
|  | **CON** | **WD diet** | **FOH diet** |
| Total energy, Kcal/100g | 386 | 476 | 476 |
| Protein contributed energy, % | 20 | 12 | 12 |
| Carbohydrate contributed energy, % | 70 | 43 | 43 |
| Fat contributed energy, % | 10 | 45 | 45 |
| Basal chow, g | 100 | 78 | 78 |
| Lard, g | / | 20 | 10 |
| Fish oil, g | / | / | 10 |
| Cholesterol, g | / | 2 | 2 |
|  |  |  |  |
| **Fatty acids profile (%of total fatty acid)** |  |  |  |
| C14:0 | 0.16 | 1.26 | 4.09 |
| C16:0 | 15.56 | 25.11 | 23.12 |
| C18:0 | 29.2 | 20.06 | 12.54 |
| **∑ Saturated FAs** | **44.91** | **46.43** | **39.75** |
| C16:1 n-7 | 1.09 | 1.86 | 5.18 |
| C18:1 n-9 | 10.03 | 32.47 | 27.15 |
| **∑ Monounsaturated FAs** | **11.12** | **34.34** | **32.33** |
| C16:3 n-3 | nd | nd | 0.64 |
| C16:4 n-3 | 2.31 | 0.33 | 1.21 |
| C18:3 n-3 | 3.61 | 1.08 | 0.75 |
| C18:4 n-3 | tr | nd | 1.37 |
| C20:5 n-3 | nd | tr | 8.11 |
| C22:5 n-3 | nd | nd | 0.94 |
| C22:6 n-3 | nd | nd | 5.75 |
| **∑ n-3 Polyunsaturated FAs** | **5.92** | **1.41** | **18.78** |
| C18:2 n-6 | 38.02 | 17.67 | 8.6 |
| C20:4 n-6 | tr | 0.15 | 0.54 |
| **∑ n-6 Polyunsaturated FAs** | **38.02** | **17.82** | **9.14** |

^a^ Some fatty acids, of which the contents are minor, trace amountor not detected, such as C22:0, C24:0, C14:1, C20:2n−6, and C20:3n−6, were not listed in the table. tr: trace; nd: non-detected.

**Table S2**. Sequence of rat primers (5’-3’).

| Gene | forward primer | reverse primer |
| --- | --- | --- |
| *Hmgcr* | GTGGGACCAACCTTCTACCTC | GTGCATTTTCTCCAGGATTGTCT |
| *Cyp7a1* | GTGCATTTTCTCCAGGATTGTCT | AGGCATACATCCCTTCCGTGA |
| *Fdps* | TCAGTGTCTGCTACGAGCC | TCGTACTTGAAGAACACACTCCG |
| *Abcg5* | CGCAGGAACCGCATTGAAA | TGTCGAAGTGGTGGAAGAGCT |
| *Abcg8* | CGTCAGATTTCCAATGACTTCCG | TCCGTCCTCCAGTTCATAGTACA |
| *Cd36* | GAGGTCCTTACACATACAGAGTTCGTT | ACAGACAGTGAAGGCTCAAAGATG |
| *Acaa1b* | CTGTCTGAGAGAGGCAACCC | TTCTCCGAGGTTATCCCCAT |
| *Gapdh* | GTATGACTCTACCCACGGCAAGT | TTCCCGTTGATGACCAGCTT |
| *Cd44* | AAGAGGAGACCCCACATGCTAC | CCTGCCATTCATTCTCAAACCACT |
| *Fasn* | GGATGTCAACAAGCCCAAGTA | TTACAGAGGAGAAGGCCACAA |
| *Sema4a* | CAAGACTGACATCTTCCTACGC | TCCTCAAAGAAGAAATAGACGACC |
| *Socs2* | AGCAGTTTGACAGCGTGGTT | AAAGGTGAACAGTCCCATTCC |
| *Srebf1* | GATGCGGCTGTCGTCTACCAT | CCACGTAGATCTCTGCCAGTGTT |
| *Mcp1* | ATGCAGGTCTCTGTCACG | AGTATGACAGAGAACTAG |
| *Scd1* | GGATGTTCTCCCGAGATTGA | TGTTCGTCAGCACCTTCTTG |
|  |  |  |
|  |  |  |
| *Insig2* | GACGGATGTGTTGAAGGATTTCT | TGGACTGAAGCAGACCAATGTC |
|  |  |  |
|  |  |  |
|  |  |  |
| *Pcsk9* | TGGCTGCATGACATTGCTTCTC | GCACTGGAGAACCACACAGG |
| *Per2* | CACCCTGAAAAGAAAGTGCGA | CAACGCCAAGGAGCTCAAGT |
| *Per3* | ATAGAACGGACGCCAGAGTGT | CGCTCCATGCTGTGAAGTTT |
| *Baml1* | CTTGCGGAATGTCACAGGCA | ACACCAGTGTTGGTTGAGAC |
|  |  |  |
|  |  |  |
|  |  |  |
|  |  |  |
|  |  |  |
|  |  |  |
|  |  |  |
|  |  |  |
|  |  |  |
|  |  |  |
|  |  |  |

**Figure S3. The weekly body weight change of experiment animals.** Values are expressed as mean±SD; n=10 in each treatment group. *, **, vs CON group; P < 0.05. 0.01, respectively.

**Table S6. Upregulated DEGs (FOH vs. WD) implicated in the enrichedgene ontology processes.**

| Cluster Enrichment Score | Category | Term | Genes | *p-v*alue |
| --- | --- | --- | --- | --- |
| 2.813 | GOTERM_BP_FAT | GO:0006631~fatty acid metabolic process | *Cyp2b2, Tyrp1, Cyp4a1, Cyp4a3, Acnat1, Ephx2, Acot2, Crat, Acot5, Acot3, Cd36, Loc299192, Acsm5* | 3.34E-05 |
|  | GOTERM_MF_FAT | GO:0016290~palmitoyl-CoA hydrolase activity | *Acnat1, Acot2, Loc299192, Acot5, Acot3* | 4.31E-04 |
|  | INTERPRO | IPR006862:Acyl-CoA thioester hydrolase/bile acid-CoA amino acid N-acetyltransferase | *Acnat1, Acot2, Loc299192, Acot5* | 0.003365 |
|  | KEGG_PATHWAY | rno01040:Biosynthesis of unsaturated fatty acids | *Acot2, Loc299192, Acot5, Acot3* | 0.045557 |
|  |  |  |  |  |
| 2.589 | SP_PIR_KEYWORDS | disulfide bond | *Cxcl1, Ly6g6d, Gcnt3, C9, Osmr, Testin, Fst, Spink1, Mme, Tesp1, Spink3, Pcolce, Ren, Pdilt, Chst15, Il13ra2, Kng1, Ntf3, Kng1l1, Lyc2, Nrxn1, Loc362795, Slit2, Lcn2, Ighg, Ddr1, Orm1, Acvr2b, Adrb1, Cd36, Colq, Igsf1, Loc500180, Ptp4a1, Pdgfrl, Wif1, Cp, Igfbp2, Car4, Klrb1c* | 1.01E-04 |
|  | GOTERM_CC_FAT | GO:0005576~extracellular region | *Cxcl1, Fras1, C9, Testin, Fst, Spink1, Loc360228, Retnlg, Tesp1, Spink3, Il34, Grem2, Pcolce, Ccl25, Serpina7, Ren, Sema3c, Lbp, Ltb, Kng1, Rgd1566380, Ntf3, Kng1l1, Slit2, Lcn2, Orm1, Colq, Igsf1, Pdgfrl, Lamc2, Wif1, Cp, Igfbp2* | 4.06E-04 |
|  | SP_PIR_KEYWORDS | Secreted | *Cxcl1, C9, S100a8, Testin, Fst, Spink1, Tesp1, Spink3, Il34, Pcolce, Serpina7, Ren, Lbp, Kng1, Rgd1566380, Ntf3, Kng1l1, Slit2, Lcn2, Orm1, Igsf1, Pdgfrl, Wif1, Cp, Igfbp2* | 0.001456 |
|  | SP_PIR_KEYWORDS | signal | *Cxcl1, Ly6g6d, C9, Osmr, Testin, Fst, Spink1, Retnlg, Tesp1, Spink3, Il34, Pcolce, Serpina7, Ren, Pdilt, Lbp, Il13ra2, Kng1, Rgd1566380, Ntf3, Kng1L1, Lyc2, Nrxn1, Slit2, Lcn2, Tns4, Orm1, Ddr1, Acvr2b, Tlcd1, Colq, Igsf1, Pdgfrl, Wif1, Cp, Igfbp2, Car4* | 0.008029 |
| 2.549 | INTERPRO | IPR017972:Cytochrome P450, conserved site | *Cyp3a18, Cyp2b2, Cyp4a1, Cyp1a1, Cyp4f18, Cyp4a3, Cyp2c12, Cyp3a9* | 6.35E-05 |
|  | KEGG_PATHWAY | rno00830:Retinol metabolism | *Cyp3a18, Cyp2b2, Cyp4a1, Cyp1a1, Cyp4a3, Cyp2c12, Cyp3a9* | 1.75E-04 |
|  | GOTERM_MF_FAT | GO:0008395~steroid hydroxylase activity | *Cyp3a18, Cyp2b2, Cyp1a1, Cyp2c12, Cyp3a9* | 2.35E-04 |
|  | COG_ONTOLOGY | Secondary metabolites biosynthesis, transport, and catabolism | *Cyp3a18, Cyp2b2, Cyp4a1, Cyp1a1, Cyp4f18, Cyp4a3, Cyp2c12, Cyp3a9* | 3.09E-04 |
|  | GOTERM_CC_FAT | GO:0005792~microsome | *Kng1, Cyp3a18, Cyp2b2, Cd36, Adrb1, Cyp4a1, Cyp1a1, Cyp4f18, Cyp4a3, Cyp2c12, Cyp3a9, Por* | 6.05E-04 |
|  | GOTERM_CC_FAT | GO:0019898~extrinsic to membrane | *Cyp3a18, Cyp2b2, Cap2, Cyp4a1, Chmp4c, Cyp4a3, Tgm1, Rgs7, Cyp2c12, Cyp3a9, Syt17, Por* | 0.00229 |
|  | GOTERM_MF_FAT | GO:0009055~electron carrier activity | *Steap4, Cyp3a18, Cyp2b2, Cyp4a1, Cyp1a1, Cyp4f18, Cyp4a3, Cyp2c12, Cyp3a9, Por* | 0.002401 |
|  | GOTERM_MF_FAT | GO:0016712~oxidoreductase activity, acting on paired donors, with incorporation or reduction of molecular oxygen, reduced flavin or flavoprotein as one donor, and incorporation of one atom of oxygen | *Cyp3a18, Cyp2b2, Cyp1a1, Cyp2c12, Cyp3a9* | 0.002673 |
|  | GOTERM_CC_FAT | GO:0005783~endoplasmic reticulum | *Kcnma1, Cyp2b2, Cyp3a18, Cyp4a1, Cyp1a1, Ntf3, Cyp4a3, Nt5c1b, Mal, Lyc2, Crat, Por, Cyp4f18, Ptp4a1, Pdilt, Cyp2c12, Cyp3a9, Car4* | 0.02248 |
|  | KEGG_PATHWAY | rno00140:Steroid hormone biosynthesis | *Cyp3a18, Cyp1a1, Hsd17b2, Cyp3a9* | 0.023105 |
| 2.131 | GOTERM_BP_FAT | GO:0008544~epidermis development | *Fras1, Ddr1, Ntf3, Fst, Tgm1, Snai1, Ltb* | 0.00221 |
| 2.047 | GOTERM_BP_FAT | GO:0006954~inflammatory response | *Kng1, Cxcl1, Nfkbiz, Orm1, C9, S100a8, Kng1l1, Ephx2, Vnn1, Lbp* | 0.00209 |
|  | GOTERM_BP_FAT | GO:0006952~defense response | *Kng1, Cxcl1, Nfkbiz, Orm1, C9, S100a8, Kng1l1, Ephx2, Vnn1, Bcl3, Lyc2, Lbp* | 0.014946 |
|  | SP_PIR_KEYWORDS | acute phase | *Kng1, Orm1, Kng1l1* | 0.034255 |
|  | GOTERM_BP_FAT | GO:0009611~response to wounding | *Kng1, Cxcl1, Nfkbiz, Orm1, C9, S100a8, Cyp1a1, Kng1l1, Ephx2, Vnn1, Lbp* | 0.049663 |
| 1.861 | GOTERM_BP_FAT | GO:0031667~response to nutrient levels | *Abcg8, Cd36, Serpina7, Cyp1a1, Hsd17b2, Socs3, Sds, Pik3c3, Cp, Runx1, Igfbp2* | 0.003747 |
|  | GOTERM_BP_FAT | GO:0009991~response to extracellular stimulus | *Abcg8, Cd36, Serpina7, Cyp1a1, Hsd17b2, Socs3, Sds, Pik3c3, Cp, Runx1, Igfbp2* | 0.006035 |
|  | GOTERM_BP_FAT | GO:0033189~response to vitamin A | *Serpina7, Cyp1a1, Hsd17b2, Runx1, Igfbp2* | 0.011129 |
| 1.763 | GOTERM_BP_FAT | GO:0007369~gastrulation | *Acvr2b, Nodal, Hira, Cml5, Epha2, Slit2* | 0.001994 |
|  | GOTERM_BP_FAT | GO:0007498~mesoderm development | *Acvr2b, Tbx3, Nodal, Epha2, Slit2* | 0.004564 |
|  | GOTERM_BP_FAT | GO:0048729~tissue morphogenesis | *Fras1, Ddr1, Tbx3, Nodal, Fst, Sema3c, Snai1, Epha2, Slit2* | 0.009548 |
| 1.684 | GOTERM_MF_FAT | GO:0046872~metal ion binding | *Kcnj16, Steap4, Pdp2, Cyp4a1, Slc13a5, S100a8, Cyp4a3, Rnf186, Spink1, Spink3, Dnase1l2, Pik3c3, Trim7, Caln1, Cyp2c12, Adcy10, Cyp3a9, Cib2, Kcnma1, Cyp3a18, Cyp1a1, Pdxp, Loc100125361, Nrxn1, Por, Wee1, Acvr2b, Dsg1b, Trim36, Car4, Cyp2b2, Tyrp1, Nt5c1b, Trim13, Mme, Mt1a, Nr1d1, Dgkg, Tgm1, Zc3h12a, Slc30a2, Lmln, Klf16, Ephx2, Snai1, P2rx5, Cyp4f18, Mt2a, Cacna1h, Cp, Acsm5, Zbtb8a* | 0.010378 |
|  | GOTERM_MF_FAT | GO:0046914~transition metal ion binding | *Cyp2b2, Steap4, Tyrp1, Pdp2, Cyp4a1, Cyp4a3, Trim13, Rnf186, Mme, Dnase1l2, Mt1a, Nr1d1, Dgkg, Trim7, Pik3c3, Zc3h12a, Slc30a2, Cyp2c12, Adcy10, Cyp3a9, Lmln, Cyp3a18, Cyp1a1, Klf16, Loc100125361, Snai1, Por, P2rx5, Acvr2b, Trim36, Cyp4f18, Cp, Car4, Zbtb8a* | 0.037608 |
|  |  |  |  |  |
| 1.625 | GOTERM_BP_FAT | GO:0042311~vasodilation | *Kcnma1, Kng1, Adrb1, Kng1l1* | 0.006125 |
|  | GOTERM_BP_FAT | GO:0003013~circulatory system process | *Kcnma1, Kng1, Adrb1, Ren, Kng1l1, Ephx2, Nkx2-1* | 0.015666 |
|  | GOTERM_BP_FAT | GO:0050880~regulation of blood vessel size | *Kcnma1, Kng1, Adrb1, Kng1L1* | 0.025596 |
| 1.323 | GOTERM_BP_FAT | GO:0003013~circulatory system process | *Kcnma1, Kng1, Adrb1, Ren, Kng1l1, Ephx2, Nkx2-1* | 0.015666 |

**Table S7. Downregulated DEGs (FOH vs. WD) implicated in the enrichedgene ontology processes.**

| Cluster Enrichment Score | Category | Term | Genes | *p-v*alue |
| --- | --- | --- | --- | --- |
| 2.751 | GOTERM_BP_FAT | GO:0009719~response to endogenous stimulus | *Srebf1, Adcy7, Snrpn, Fads1, Socs1, Il10, Slc34a2, H19, Mgst3, Prkar2b, Gstm3, Cdkn1a, Insig2, Pcsk9, Cyp4a8, Hsd11b2, Lox, Col1a1, Pik3r3, Akr1c1* | 8.94E-04 |
|  | GOTERM_BP_FAT | GO:0048545~response to steroid hormone stimulus | *Srebf1, Gstm3, Cdkn1a, Socs1, Hsd11b2, Cyp4a8, Col1a1, Lox, Il10, Akr1c1, H19, Slc34a2* | 0.004223 |
|  | GOTERM_BP_FAT | GO:0043434~response to peptide hormone stimulus | *Srebf1, Insig2, Fads1, Socs1, Hsd11b2, Pcsk9, Col1a1, Pik3r3, Il10, H19* | 0.005991 |
|  | GOTERM_BP_FAT | GO:0032868~response to insulin stimulus | *Srebf1, Insig2, Fads1, Hsd11b2, Pcsk9, Pik3r3, Il10* | 0.014501 |
|  |  |  |  |  |
| 2.362 | SP_PIR_KEYWORDS | glycoprotein | *Steap3, Lypd1, Casr, Slc44a3, Slc6a20, Cd8b, Mmp9, Slc6a4, Rprm, Gdnf, Il10, Sctr, Ednra, Clec4e, Slc22a3, Col12a1, Sema3a, Lox, Izumo1, Myc, Odz2, Dpep1, Scn10a, Lair1, Ptger2, Cd3d, Ptprn2, Colec12, Slc34a2, Dnase2b, Inhbb, Inhba, Egflam, Adam18, Col1a1, Enpp7, Adora2b, Loc363060, St8Sia1, Cx3cl1, Hmmr, Pcsk9, Mfap3l, Slco3a1, Gpnmb, Thbs4, Olr59, Il2ra, Olr1, Chi3l1, Nlgn3, Cacna2d3, Ace2, Cd200, Slc14a2* | 5.79E-05 |
|  | UP_SEQ_FEATURE | transmembrane region | *Steap3, Casr, Slc44a3, Slc6a20, Cd8b, Cldn5, Slc6a4, Rprm, Sctr, Ednra, Atp2b2, Ptgis, Insig2, Elovl5, Clec4e, Slc22a3, Izumo1, Odz2, Scn10a, Lair1, Ptger2, Cd3d, Ptprn2, Tmem38a, Colec12, Stx1b, Slc34a2, Cldn1, Adam18, Gpam, Fxyd2, Enpp7, Adora2b, Loc363060, Cx3cl1, Mfap3l, Slco3a1, Gpnmb, Srebf1, Scd1, Olr59, Il2ra, Olr1, Fads1, Atp1a3, Fads2, Nlgn3, Cacna2d3, Slc16a3, Ace2, Syt15, Cyb5d2, Cd200, Slc14a2* | 6.39E-04 |
|  | UP_SEQ_FEATURE | signal peptide | *Lypd1, Casr, Enpp7, Loc363060, Cd8B, Mmp9, Btc, Cx3Cl1, Rt1-Doa, Gdnf, Il10, Sctr, Apoa4, Ednra, Resp18, Pcsk9, Sema3A, Mfap3L, Izumo1, Lox, Gpnmb, Dpep1, Thbs4, Lair1, Il2Ra, Cd3D, Ptprn2, Chi3L1, Nlgn3, Cacna2D3, Dnase2B, Inhbb, Inhba, Egflam, Ace2, Fcrla, Col1A1, Cd200* | 0.011277 |
|  | SP_PIR_KEYWORDS | membrane | *Steap3, Lypd1, Casr, Lzts1, Slc44a3, Slc6a20, Cd8b, Cldn5, Slc6a4, Rprm, Sctr, Ednra, Atp2b2, Ptgis, Insig2, Clec4e, Elovl5, Cyp4a8, Slc22a3, Izumo1, Odz2, Dpep1, Scn10a, Lair1, Ptger2, Cd3d, Ptprn2, Tmem38a, Colec12, Stx1b, Slc34a2, Ccr7, Cldn1, Adam18, Gpam, Unc13a, Fxyd2, Enpp7, Adora2b, Loc363060, St8Sia1, Cx3cl1, Dgkb, Slco3a1, Mfap3l, Gpnmb, Srebf1, Scd1, Olr59, Il2ra, Olr1, Rt1-Ce12, Fads1, Rt1-Ce13, Atp1a3, Fads2, Nlgn3, Cacna2d3, Rgs14, Slc16a3, Cadps, Ace2, Syt15, Cyb5d2, Cd200, Slc14a2* | 0.015583 |
|  |  |  |  |  |
| 2.311 | GOTERM_CC_FAT | GO:0005578~proteinaceous extracellular matrix | *Wnt2, Egflam, Wnt5B, Lgals1, Mmp9, Rgd1565947, Col12A1, Postn, Col1A1, Lox, Thbs4* | 0.001781 |
|  | GOTERM_CC_FAT | GO:0005576~extracellular region | *Wnt5b, Mmp9, Cxcl9, Postn, Cx3cl1, Gdnf, Il10, Apoa4, Wnt2, Ptgis, Resp18, Rgd1565947, Pcsk9, Col12a1, Lox, Sema3A, Thbs4, Olr1, Lgals1, Chi3l1, Vash1, Dnase2b, Inhbb, Inhba, Egflam, Cxcl14, Cxcl13, Cpxm1, Ace2, Fcrla, Col1a1* | 0.008708 |
|  |  |  |  |  |
| 2.148 | GOTERM_MF_FAT | GO:0030246~carbohydrate binding | *Olr1, Lgals1, Chi3l1, Colec12, Postn, Pfkm, Hmmr, Egflam, Pygm, Clec4e, Gck, Gfpt2, Clec7a, Gpnmb* | 0.0011 |
|  | GOTERM_MF_FAT | GO:0005529~sugar binding | *Clec4e, Olr1, Pygm, Gck, Lgals1, Gfpt2, Chi3l1, Colec12, Clec7a, Pfkm* | 0.003743 |
|  |  |  |  |  |
| 2.025 | GOTERM_BP_FAT | GO:0009991~response to extracellular stimulus | *Apoa4, Srebf1, Cdkn1a, Gck, Adora2b, Fads1, Mmp9, Hsd11b2, Slc22a3, Pcsk9, Col1a1, H19* | 0.005539 |
|  | GOTERM_BP_FAT | GO:0043434~response to peptide hormone stimulus | *Srebf1, Insig2, Fads1, Socs1, Hsd11b2, Pcsk9, Col1a1, Pik3r3, Il10, H19* | 0.005991 |
|  | GOTERM_BP_FAT | GO:0031667~response to nutrient levels | *Apoa4, Srebf1, Gck, Fads1, Mmp9, Hsd11b2, Slc22a3, Pcsk9, Col1a1, H19* | 0.025232 |
|  |  |  |  |  |
| 1.991 | GOTERM_CC_FAT | GO:0005578~proteinaceous extracellular matrix | *Wnt2, Egflam, Wnt5b, Lgals1, Mmp9, Rgd1565947, Col12a1, Postn, Col1a1, Lox, Thbs4* | 0.001781 |
|  | GOTERM_BP_FAT | GO:0043062~extracellular structure organization | *Atp2b2, Egflam, Mmp9, Postn, Nlgn3, Ky, Col1a1, Lox* | 0.004555 |
|  |  |  |  |  |
| 1.863 | GOTERM_BP_FAT | GO:0008610~lipid biosynthetic process | *Scd1, Srebf1, Chka, Ptgis, Elovl5, Insig2, Fads1, Fads2, Gpam, Fabp5* | 0.024466 |
|  | GOTERM_BP_FAT | GO:0006633~fatty acid biosynthetic process | *Scd1, Ptgis, Elovl5, Fads1, Fads2* | 0.026581 |
|  |  |  |  |  |
| 1.846 | GOTERM_MF_FAT | GO:0042802~identical protein binding | *Bcat1, Chka, Snrpn, Lgals1, Slc6a4, Cldn5, Pfkm, Gdnf, Abcg4, Apoa4, Inhbb, Inhba, Gstm3, Pygm, Anks6, Cldn1, Pcsk9, Col1a1, Unc13a* | 0.001871 |
|  | GOTERM_MF_FAT | GO:0042803~protein homodimerization activity | *Inhbb, Apoa4, Chka, Pygm, Lgals1, Anks6, Slc6a4, Gdnf, Abcg4, Unc13a* | 0.039029 |
|  | GOTERM_MF_FAT | GO:0046983~protein dimerization activity | *Chka, Batf2, Cd3d, Lgals1, Slc6a4, Gdnf, Abcg4, Apoa4, Inhbb, Inhba, Pygm, Anks6, Myc, Unc13a* | 0.039542 |
|  |  |  |  |  |
| 1.843 | GOTERM_BP_FAT | GO:0031668~cellular response to extracellular stimulus | *Srebf1, Cdkn1a, Gck, Adora2b, Fads1, Pcsk9* | 0.002776 |
|  | GOTERM_BP_FAT | GO:0009267~cellular response to starvation | *Srebf1, Gck, Fads1, Pcsk9* | 0.010737 |
|  | GOTERM_BP_FAT | GO:0032868~response to insulin stimulus | *Srebf1, Insig2, Fads1, Hsd11b2, Pcsk9, Pik3r3, Il10* | 0.014501 |
|  |  |  |  |  |
| 1.523 | GOTERM_MF_FAT | GO:0008009~chemokine activity | *Cxcl14, Cxcl13, Cxcl9, Cx3Cl1* | 0.010502 |
|  | GOTERM_MF_FAT | GO:0005125~cytokine activity | *Inhba, Cxcl14, Cxcl13, Cxcl9, Cx3cl1, Il10* | 0.020695 |
|  | GOTERM_BP_FAT | GO:0032101~regulation of response to external stimulus | *Gpx2, Ednra, Il2ra, Adora2b, Cxcl9, Cx3cl1, Il10* | 0.033155 |
|  | KEGG_PATHWAY | rno04062:Chemokine signaling pathway | *Ccr7, Cxcl14, Adcy7, Cxcl13, Ncf1, Cxcl9, Cx3cl1, Pik3r3* | 0.03436 |
